# Valorisation of Lignocellulosic Wastes, the Case Study of Eucalypt Stumps Lignin as Bioadsorbent for the Removal of Cr(VI)

**DOI:** 10.3390/molecules27196246

**Published:** 2022-09-22

**Authors:** Ana Lourenço, Dragana Kukić, Vesna Vasić, Ricardo A. Costa, Mirjana Antov, Marina Šćiban, Jorge Gominho

**Affiliations:** 1 Centro de Estudos Florestais, Instituto Superior de Agronomia, Universidade de Lisboa, Tapada da Ajuda, 1349-17 Lisboa, Portugal; 2 Faculty of Technology Novi Sad, University of Novi Sad, Bulevar Cara Lazara 1, 21 000 Novi Sad, Serbia

**Keywords:** adsorption, *Eucalyptus*, kraft lignin, chromium, analytical pyrolysis, biorefinery

## Abstract

The main objective of this work was to assess *Eucalyptus globulus* lignin as an adsorbent and compare the results with kraft lignin, which has previously been demonstrated to be an effective adsorbent. Eucalypt lignin was extracted (by the dioxane technique), characterised, and its adsorption properties for Cr(VI) ions were evaluated. The monomeric composition of both types of lignin indicated a high content of guaiacyl (G) and syringyl (S) units but low content of *p*-hydroxyphenyl (H), with an H:G:S ratio of 1:50:146 (eucalypt lignin) and 1:16:26 (kraft lignin), as determined by Py-GC/MS. According to elemental analysis, sulphur (2%) and sodium (1%) were found in kraft lignin, but not in eucalypt lignin. The adsorption capacity of the eucalypt lignin was notably higher than the kraft lignin during the first 8 h, but practically all the ions had been absorbed by both the eucalypt and kraft lignin after 24 h (93.4% and 95%, respectively). Cr(VI) adsorption onto both lignins fitted well using the Langmuir adsorption isotherm model, with capacities of 256.4 and 303.0 mg/g, respectively, for eucalypt and kraft. The study’s overall results demonstrate the great potential of eucalypt lignin as a biosorbent for Cr(VI) removal from aqueous solutions.

## 1. Introduction

Lignin is the second main constituent of lignocellulosic biomass and is the second most abundant biopolymer in nature [1]. Its structural and chemical properties vary, depending on the type of lignocellulosic material, growing conditions, and the isolation process [2]. Nowadays, a significant amount of lignin is produced, with worldwide production ranging from 40 to 50 million tons per year, obtained as a by-product from pulping and biofuel production [3], but less than 2% has been purified (mostly from lignosulphonates or kraft lignin) for further applications [4]. Other lignins, such as dioxane and Björkman, are used to evaluate the lignin structure and composition because these are closer to the in situ lignin. Lignin valorisation through its employment in various materials (composites, additives, phenolic resins), has been actively researched in recent years for use as fuel or for the synthesis of aromatic compounds [3,5,6].

Furthermore, lignin has been reported as a promising eco-friendly adsorbent to remove heavy metals, due to its abundance, low cost, and physicochemical properties [7,8,9]. This complex heteropolymer contains various functional groups, such as carboxylic and phenolic groups, representing active sites for metal ion binding. Lignin presents a significant advantage in water treatment as it does not impose environmental issues, due to its insolubility in water and because it is entirely biodegradable. The possibility of metal recovery from lignin by acidification is followed by precipitation [10], and heavy metal adsorption onto lignins of various origins has been documented in the literature [1,11,12,13,14,15]. More recently, lignin modifications have been made to improve its adsorption capacity [16,17]. 

The presence of hexavalent chromium in the effluents of different industries (e.g., leather tanning, cement, metallurgical and wood preservation) can cause severe environmental and health problems due to its persistence and carcinogenicity [10]. These effluents need to be treated to acceptable levels before discharge. Different procedures can be used (e.g., chemical precipitation, lime coagulation, ion exchange, membrane techniques, or adsorption by activated carbon [18]); however, these methods face disadvantages, such as insufficient efficacy, a significant amount of toxic sludge or high costs, and more efficient and economically-friendly procedures are therefore required. Adsorption is considered one of the best conventional methods due to its simplicity, efficiency, and the possibility of adsorbent regeneration [18]. A wide range of lignocellulosic materials have been investigated for this purpose and proven efficient in heavy metal removal from aqueous solutions (e.g., *Eucalyptus globulus* and *Cupressus lusitanica*) [19,20]. 

*E. globulus* stumps (the basal part of the tree) represent about 25% of aboveground biomass, amounting to approximately 40–60 tons per ha, and are chemically characterised by a high percentage of extractives (15.1%), holocellulose (67.0%), and lignin content (24.8%) [21]. Nowadays, eucalypt stumps are used by the pulp and paper industries, due to the scarcity of feedstock for this industry and as reported in the literature [22,23]. Other possible uses have also been explored: the production of pellets for energy purposes, e.g., syngas with high quality [24], and the extraction of compounds with biological activities [25]. Different stump valorisation pathways, such as those based on lignin, are possible but still need to be investigated; for example, lignin could be used as an adsorbent in water treatment. The practical application of this lignin would contribute to the valorisation of stumps under the biorefinery and circular economy concepts and the development of sustainable wastewater treatment technologies. Available literature also provides limited information regarding the adsorption properties of the lignin from *E. globulus* stumps isolated by the dioxane method. Thus, the goals of this study were to: (1) isolate lignin from *E. globulus* stumps (referred to here as eucalypt stump lignin) using the dioxane method (to obtain lignin that is closer to lignin in the matrix and with a high yield) [26], (2) characterise it using some of the available techniques, and (3) evaluate its use for Cr(VI) ion removal from aqueous solution. The adsorption properties of the lignin were assessed through equilibrium and kinetic studies and compared with kraft lignin, which has been extensively studied for the same purpose.

## 2. Results and Discussion

### 2.1. Lignin Characterisation

**Figure 1:**
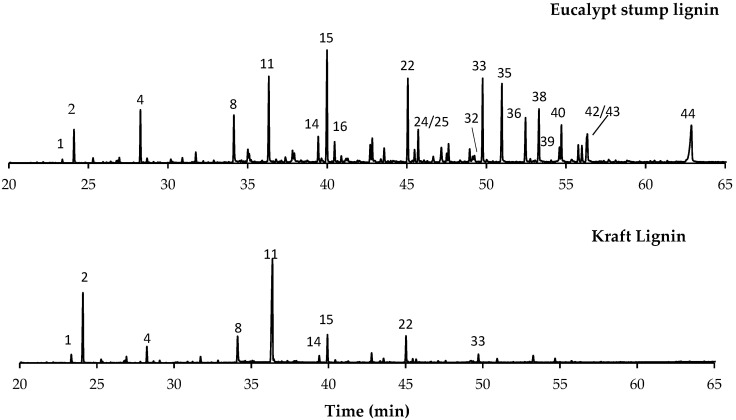
Pyrograms of the lignins from eucalypt stump and kraft lignin from poplar and beech (50:50). Identified compounds: 1—phenol; 2—guaiacol; 4—4-methylguaiacol; 8—4-vinylguaiacol; 11—syringol; 14—*trans* isoeugenol; 15—4-methylsyringol; 16—vanillin; 22—4-vinylsyringol; 24—4-allylsyringol; 25—4-propylsyringol; 32—1,6-anhydro-β-D-glucopyranose; 33—*trans*-4-propenylsyringol; 35—syringaldehyde; 36—homosyringaldehyde; 38—acetosyringone; 39—*trans* coniferaldehyde; 40—syringylacetone; 42—syringyl vinyl ketone; 43—sinapyl alcohol isomer; 44—*trans* sinapaldehyde.

The pyrolysis results of the isolated lignins are presented in Table 1, and the corresponding pyrograms are shown in Figure 1. This analysis demonstrated that eucalypt stump lignin was characterised by a predominant composition of S units (62%), followed by G units (21%) and a small number of H units (0.4%). In the case of kraft lignin, this distribution was 50%, 31%, and 2%, respectively. The main compounds identified in eucalypt stump lignin were: 4-methylsyringol (peak **15**, 8.5%), syringol (peak **11**, 6.5%), syringaldehyde (peak **35**, 6.2%), 4-vinylsyringol (peak **22**, 6.1%), acetosyringone (peak **38**, 4.2%), 4-vinylguaiacol (peak **8**, 3.6%), homosyringaldehyde (peak **36**, 3.6%), and 4-methylguaiacol (peak **4**, 3.5%). 

Kraft lignin pyrolysis induced a lower production of compounds when compared with eucalypt stump lignin (Figure 1), with a predominance of syringol (peak **11**, 30.7%) and guaiacol (peak **2**, 14.2%). In contrast, other monomers, such as 4-vinylguaiacol (peak **8**, 6.9%), 4-methylsyringol (peak **15**, 5.4%), and 4-vinylsyringol (peak **22**, 5.2%), were formed in amounts lower than 10% (Table 1 and Figure 1). These differences could be associated with the species and the isolation method. For example, the lignin mass obtained by the dioxane method is about 60–65% of the Klason lignin from eucalypt stumps; however, a higher value (80%, based on Klason lignin) was reported by Evtuguin et al. for eucalypt wood [26]. The dioxane method allows the preservation of the lignin structure, and the monomeric composition is quite similar to the lignin structure of the wood matrix, i.e., close to pristine lignin.

The monomeric H:G:S composition was 1:50:146 and 1:16:26 for eucalypt stump lignin and kraft lignin, respectively, whereas the S/G ratios were 2.9 and 1.6 (Table 1). It was also possible to verify by Py-GC/MS that both lignins were highly pure, with a low amount of sugars, since the presence of levoglucosan (peak **32**), a derivative of cellulose during pyrolysis [27], only appeared in amounts of 0.6% and 0.3% in eucalypt stump and kraft lignins, respectively. Overall, the composition of these isolated lignins shows that S units are predominant over the G and H units, which is in accordance with the literature for hardwood lignin [2].

**Figure 2:**
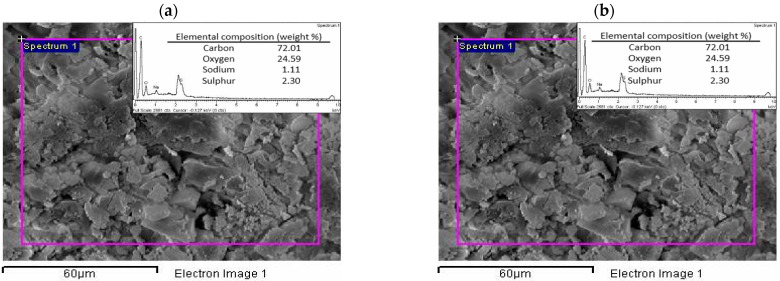
Energy-dispersive X-ray spectroscopy (EDX) spectra of the (**a**) eucalypt stump and (**b**) kraft lignin.

The elemental composition of the eucalypt stump lignin and kraft lignin is presented in Table 2. It can be seen that both lignins show similar content in their elemental composition, except that the kraft lignin contains 2.0% sulphur since it was isolated from the kraft liquor. Carbon (58% and 61%) and oxygen (36% and 33%) are the main elements present in the eucalypt stump and kraft lignins, indicating many oxygenated functional groups. The investigated lignins have a carbon content that is comparable with other types of lignin, such as Klason lignin from *E. grandis*: 58% C, 5% H, 0.1% N, 0.3% S, and 37% O [13]; alkali lignin from *E. grandis* x *E. urophylla*: 59% C, 6% H, and 35% O [28]. EDX analysis (Figure 2) showed that the kraft lignin also included sodium in its composition (1.1% weight, Figure 2b), which originates from the kraft pulping, along with the sulphur. None of these elements were detected in the eucalypt stump lignin because the lignin was directly isolated from the raw material (eucalypt stumps, Figure 2a).

**Figure 3:**
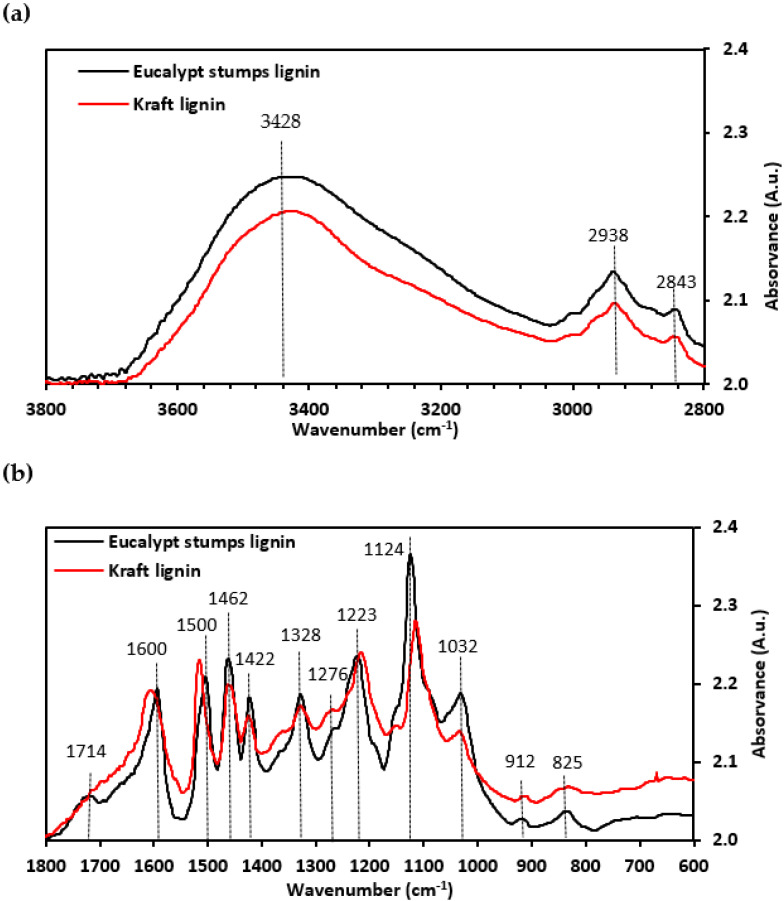
FTIR spectra of the eucalypt stump lignin and kraft lignin are depicted in the region 3800–2800 cm^−1^ (**a**) and in the region 1800-600 cm^−1^ (**b**). Measurements in absorbance units (A.u.).

FTIR was used to identify the functional groups on the surface of both investigated lignins and provide insight into their involvement in the adsorption process. The spectra in Figure 3 show the band intensities of the eucalypt stump and kraft lignins before the adsorption of Cr(VI) ions. The assignment of the bands of lignin samples was determined based on the literature [1,13,29,30,31,32]. According to Faix [31], the two lignins are GS type 4 because the band at around 1121 cm^−1^ dominates over the other bands in the fingerprint region. The eucalypt stump lignin has both S and G units, as reported elsewhere [2], as well as the investigated kraft lignin (derived from the hardwoods poplar and beech wood [33,34,35]), which was confirmed by pyrolysis. The FTIR spectra of these lignins have a broadband corresponding to the OH groups of phenolic and aliphatic structures (3428 cm^−1^). The bands centred around 2938 cm^−1^ and 2843 cm^−1^ are assigned to the C-H stretching in methoxyl, methyl, and methylene groups (Figure 3a) [29,32,34]. In the fingerprint region (Figure 3b), a band associated with the unconjugated carbonyl stretching of aldehyde/ketone groups [32,34], centred at 1714 cm^−1^, is present in the eucalypt stump lignin spectrum but does not appear in the kraft lignin spectrum. Both spectra show bands assigned to aromatic skeletal vibration (around 1600, 1500, and 1422 cm^−1^), as well as C-H bending in the methyl and methylene groups (1462 cm^−1^) and C_aryl_-O vibration in the syringyl derivatives (1328 cm^−1^) [32]. The latter was of significantly lower intensity in the kraft lignin spectra. Furthermore, both spectra show bands characteristic of the C-C and C-O stretching of the G unit (1276 and 1223 cm^−1^), aromatic C-H deformation in the syringyl ring (1124 cm^−1^) and aromatic C-H in-plane deformations in the G units and C-O deformations in primary alcohols (1032 cm^−1^). C-H bands from the aromatic ring (band out-of-plane) at 912 cm^−1^ and C-H in the aromatic ring from the S unit at 825 cm^−1^ (band out-of-plane) [34] were also observed.

**Figure 4:**
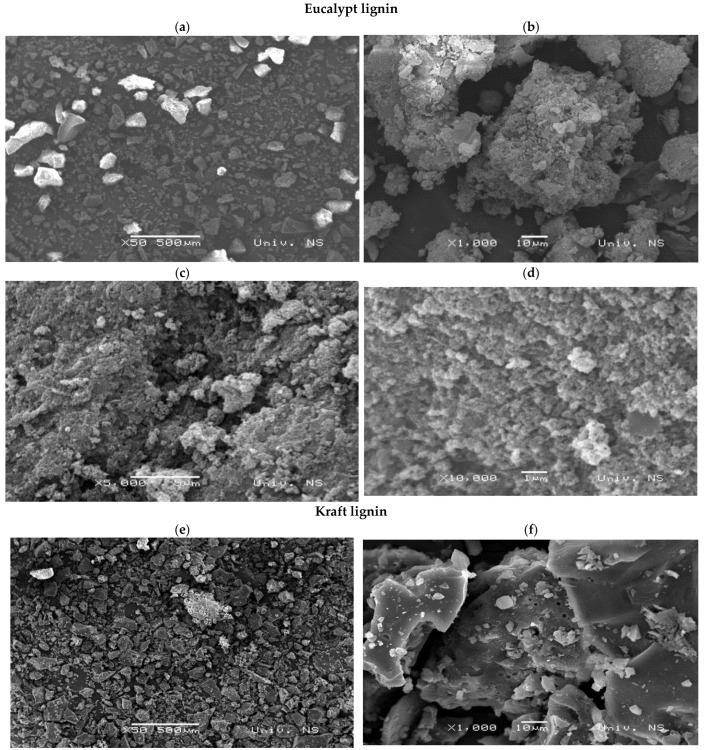

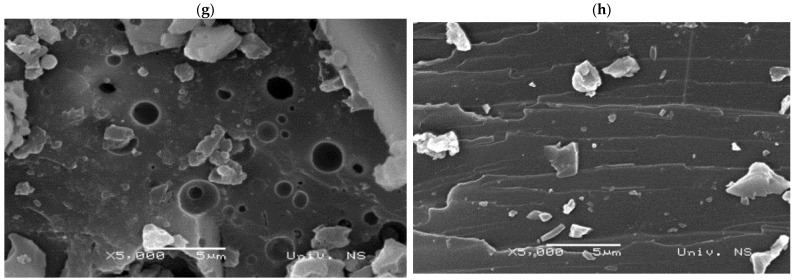
Eucalypt stump and Kraft lignins were observed by scanning electron microscopy. Magnification scales: (**a**,**e**)—×50; (**b**,**f**)—×1000; (**c**,**g**,**h**)—×5000, (**d**)—×10,000.

Figure 4 presents eucalypt stump and kraft lignins’ SEM micrographs. The eucalypt stump lignin shows uniformity regarding its highly rough surface, with cavities and no pores, and is strongly agglomerated. It has an average particle size of 136.4 µm (STDEV 68.5 µm), ranging between 64.9 and 378.6 µm. The kraft lignin particles seem more heterogeneous regarding their surface, presenting layered flat areas and many pores suitable for ion binding. The particle size of the kraft lignin is 105.9 µm on average (STDEV 68.5 µm), ranging between 17.7 and 248.5 µm. 

**Figure 5:**
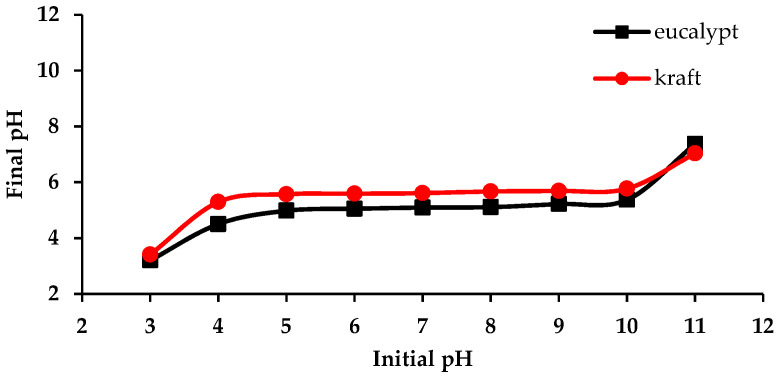
pH_pzc_ curves for the eucalypt stump lignin and kraft lignin.

The pH_pzc_ represents the pH of a solution at which the adsorbent surface is neutral, meaning that the number of positively and negatively charged functional groups is equal. This information is beneficial for evaluating new materials as adsorbents: if the pH of a solution is lower than pH_pzc_, the adsorbent surface is positively charged, and the adsorbent is more likely to adsorb anions, and vice versa [36]. Figure 5 shows that the pH_pzc_ of eucalypt stump and kraft lignins are 5.1 and 5.6, respectively, which implies that the surface of the adsorbents in solutions with a pH below the aforementioned values is positive. Since the chromium ions at pH 2 are in the form of anions, this favours their adsorption onto both types of lignin.

### 2.2. Adsorption Kinetics

**Figure 6:**
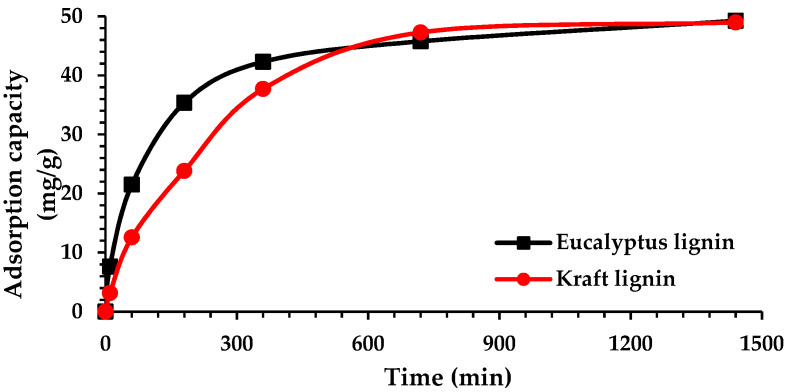
Effect of time on adsorption capacity of lignin for Cr(VI) ions (C_0_ = 50 mg/L, m = 0.1 g, V = 100 mL).

The kinetics study was conducted by varying the contact time (10 min to 24 h) of 1 g/L of lignin with a chromium solution with an initial concentration of about 50 mg/L. The results are presented in Figure 6. After 24 h (1440 min) of contact, almost all ions in the solution were removed by the eucalypt stump and kraft lignins (93.4% and 95%, respectively), reaching the adsorption capacities of 49.2 and 48.9 mg/g, respectively. However, the adsorption onto eucalypt stump lignin was faster during the first 8 h (480 min), and its adsorption capacity was notably higher than the capacity of the kraft lignin during this period (Figure 6), which may be the consequence of the different particle size distribution in the samples, as well as the other generation processes which can affect the physical structure. SEM images showed no pores on the eucalypt lignin particles, so it is possible that the adsorption occurs mainly on the outer surface and, therefore, is faster. After 360 min, about 86% of the total initial number of ions was adsorbed, which is a significant advantage in terms of its practical application in water treatment: with the reduction in contact time, the operational costs of the process would also reduce, although the use of eucalypt stump lignin compared with kraft lignin would provide a lower residual concentration. Other industrial lignins (e.g., pulping black liquor) revealed a long time (4320 min) to reach the adsorption equilibrium [15,16].

Several kinetic models fitted the experimental data shown in Figure 6, providing more information about the process rate and rate-controlling step, which can be essential for setting up an adsorber. The values of the corresponding parameters are shown in Table 3. The extremely high values of R^2^ for the pseudo-second-order model for both adsorbents (0.9995 and 0.9951) indicate that the limiting factor of the adsorption rate of Cr(VI) ions could be chemisorption, since the other two models provided a comparatively poorer fit. Similar behaviour was reported by Zhang and Zhou [15], testing industrial lignins as adsorbents for Cr(VI) and attained R^2^ > 0.995 for the pseudo-second-order model. Furthermore, the value of the pseudo-second-order rate constant, k_2_, for eucalypt lignin is significantly higher, implying faster adsorption (0.00835 g/mg min), as previously discussed. Also, the q_e_ values calculated by the pseudo-second-order model agreed very well with the experimental data. On the other hand, the rate constant of the pseudo-first-order model for kraft lignin showed a neglectful higher value indicating the same adsorption rate for both adsorbents but lower R^2^ values compared with the pseudo-second-order model.

### 2.3. Isotherm Study

**Figure 7:**
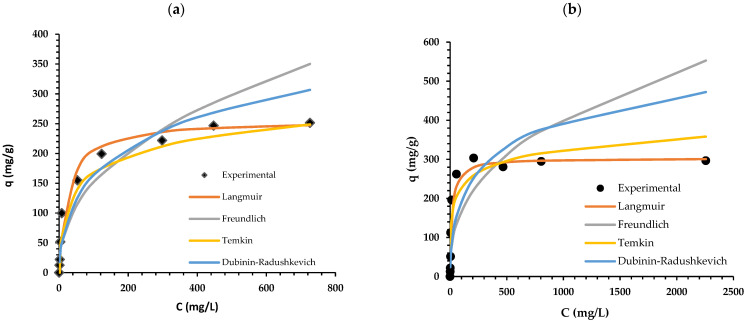
Adsorption isotherms for Cr(VI) adsorption onto eucalypt stump lignin (**a**); and onto kraft lignin (**b**).

Adsorption isotherms are mathematical models used to describe the adsorption process and express the distribution of adsorbate between the liquid and solid phases. The information gained through adsorption isotherm analyses can be helpful for the practical application of an adsorbent. This study applied some of the most commonly used models (Table 4). The experimental data were obtained by batch adsorption using Cr(VI) solutions of different initial concentrations (10, 20, 50, 100, 200, 300, 500, 700, and 1000 mg/L) and a contact time of 24 h for both types of lignin, and the results are shown in Figure 7. The adsorption capacity of eucalypt stump lignin for Cr(VI) ions was calculated using linearised isotherm models (Table 4) for fitting the experimental data. The initial chromium concentrations were chosen according to their concentrations in real effluents. Higher amounts of these ions had to be applied to the solution to reach the adsorption equilibrium, which is graphically represented by a plateau. The adsorption of chromium ions by eucalypt stump lignin can be described very well by the Langmuir (R^2^ 0.9979) and Temkin (R^2^ 0.9916) models, according to determination coefficients. In contrast, the Freundlich and Dubinin–Radushkevich models provided poor fitting (R^2^ of 0.8642 and 0.9141, respectively). Fitting by the Langmuir model resulted in a slightly higher R^2^ (0.9979), indicating that the surface of this type of lignin is homogenous, with a fixed number of identical active sites for Cr(VI) ions [15]. Furthermore, the value obtained from the equation for the maximum adsorption capacity, q_m_, was 256.4 mg/g, which is approximate to the experimentally obtained value (251.3 mg/g, Figure 7a). 

The adsorption of Cr(VI) ions by kraft lignin was poorly described by all applied models other than the Langmuir model, which gave an extremely high value for the determination coefficient (0.9997). Mohan et al. [11] reported values for the adsorption capacities of copper (137 mg/g) and cadmium ions (87 mg/g) onto kraft lignin derived from *Eucalyptus* black liquor, where both the Langmuir and Freundlich models adequately fitted the data, whereas Šćiban et al. [12] reported an adsorption capacity of 13.4 mg/g for Cr(VI) ions onto kraft lignin. Recently, published papers have described the adsorption capacities of lignin isolated from *Pinus elliotii* and *Eucalyptus grandis* to be 16.55 mg/g for zinc ions [1]. However, higher values were mentioned when using modified lignins, e.g., by introducing amino groups, which increased from 409.3 to 559.7 mg/g [16].

**Figure 8:**
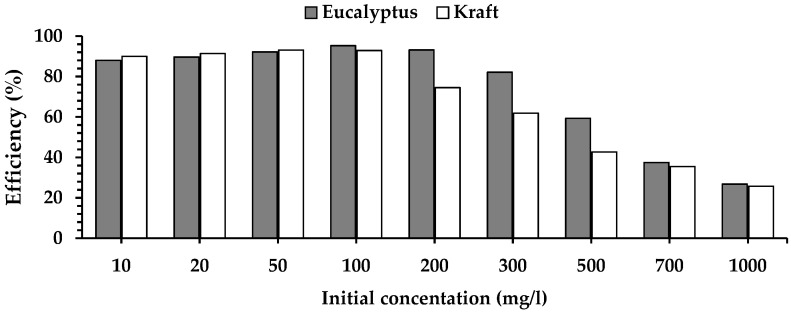
The efficiency of eucalypt stump lignin and kraft lignin in the removal of Cr(VI) at different initial concentrations.

The calculated adsorption capacity was the same as the experimentally obtained value. A dimensionless separation factor, R_L_, also indicates favourable adsorption. Both lignins showed high efficiency for removing Cr(VI) ions (Figure 8), with the kraft lignin appearing slightly more efficient at the initial concentration of 50 mg/L and lower. Surprisingly, at concentrations between 100 and 500 mg/L, the efficiency of eucalypt stump lignin increases with the increase in ion concentration, compared with kraft lignin. The good results attained by these lignins are related to the presence of functional groups such as alcoholic and phenolic hydroxyl groups and methoxyl groups that can bind metal ions [13]. 

**Figure 9:**
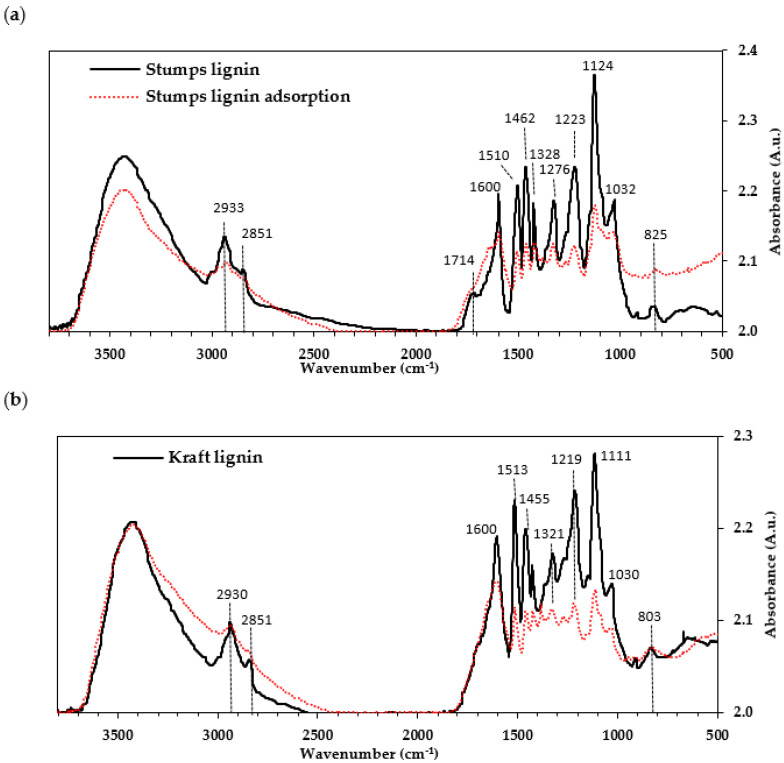
FTIR spectra of lignin samples before and after adsorption of Cr(VI) depicted in the region 500–3800 cm^−1^: eucalypt stump lignin (**a**) and kraft lignin (**b**). Measurements in absorbance units (A.u.).

The FTIR spectra of the lignins before and after the adsorption experiments show noticeable differences (Figure 9a,b). According to the FTIR analysis of the initial lignins, both present a broad band in the hydroxyl (-OH) and methoxyl groups (-OCH_3_) region (Figure 2); however, after adsorption, the eucalypt stump lignin spectrum shows a slightly broader band of lower intensity corresponding to the OH groups and almost a complete disappearance of the bands related to C-H stretching in the aromatic methoxyl group (2851 cm^−1^) and C-H stretching in the methyl and methylene groups (2933 cm^−1^). Similar trends were reported by Gupta and Mondal [41] when studying biochar as an adsorbent of Cr(VI), where the signals related to C–O, O–H, C–H, and C=O groups decreased, suggesting their participation in the adsorption of Cr(VI). This study also mentioned that the main functional groups involved in Cr(VI) adsorption were C–O and C=O [41]. Vinodhini and Das also report the involvement of bonded OH groups, C=O and C-O stretching in the Cr(VI) biosorption [42]. In our samples, a decrease in the carbonyl-stretching group (1714 cm^−1^) was observed after adsorption (Figure 9), as was also mentioned by Suksabye and Thiravetyan [43]. In addition, a decrease in the intensity of the bands between 1600 to 1000 cm^−1^ was observed, meaning that functional groups such as the methoxyl groups (OCH_3_), which have a band of 1462 cm^−1^, may participate in the adsorption of Cr(VI), as also mentioned in literature [41,42,43]. More recently, Zhang and Zhou [15] also refer to a disappearance of the bands related to CH stretching, phenolic OH, CO stretching, and carbonyl groups in the lignins after the adsorption of Cr(VI). 

Comparable behaviour was found in the FTIR spectra of the kraft lignin, as shown in Figure 9b. Decreased intensities of these bands imply their involvement in the adsorption process and contribution to the total uptake capacity. Although these lignins are of different origins, according to the FTIR spectra, the functional groups present in their structure are similar, and probably the same groups participate in metal ion binding. 

There is almost no available literature concerning the use of eucalypt stump lignin isolated by dioxane as an adsorbent. However, Mohan et al. [11] have successfully used eucalypt lignin isolated from black liquor to remove Cu(II) and Cd(II) in aqueous solutions. This achievement was related to the functional groups present in lignin, such as the phenolic groups that act as active sites for adsorption [11], which may explain the Cr(VI) ion binding ability of both lignins.

**Figure 10:**
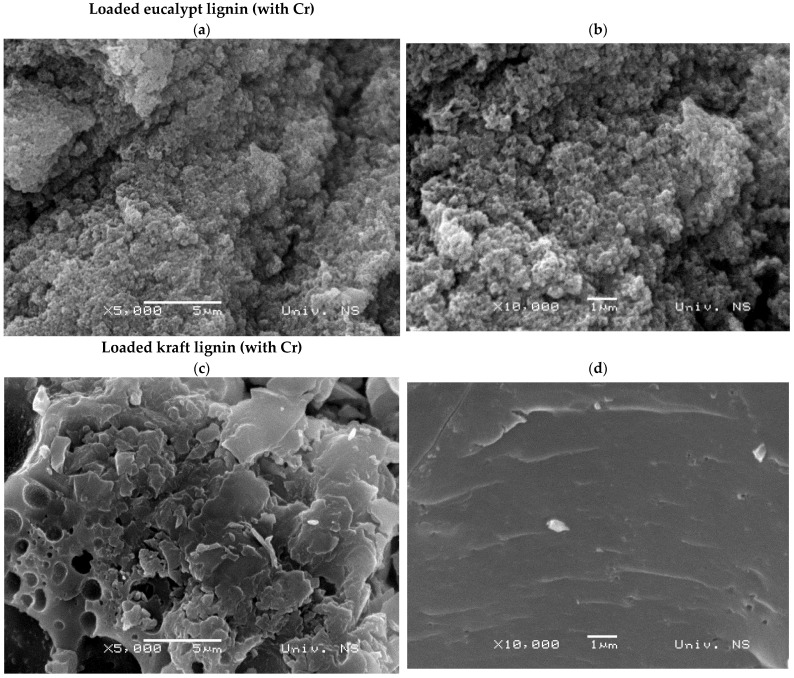
SEM images of the eucalypt stump and kraft lignin after adsorption of Cr(VI) ions. Magnification scales: (**a**,**c**)—×5000; (**b**,**d**)—×10,000.

**Figure 11:**
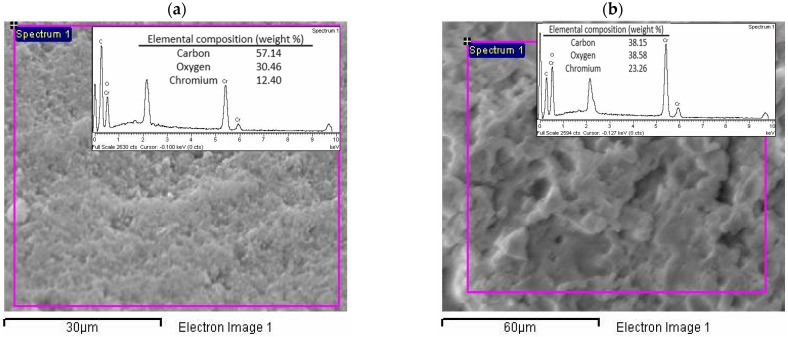
EDX spectra analysis of the eucalypt stump (**a**) and kraft lignin (**b**) after adsorption of Cr(VI) ions.

The SEM images of the lignins after adsorption showed no significant changes compared with the initial lignins, so the adsorption of chromium ions appears not to have any influence on the surface structure of the lignin (Figure 10). The corresponding elemental composition by EDX showed the presence of chromium in both lignins, but kraft lignin was able to adsorb twice the amount (23.3% vs. 12.4%, Figure 11). The EDX spectrum of the kraft lignin does not show the presence of sulphur and sodium, which suggests the possibility of ion exchange as an adsorption mechanism.

## 3. Materials and Methods

### 3.1. Eucalypt Stump Lignin

Industrial chips from *E. globulus* stumps were provided by Altri-Celtejo, a Portuguese Pulp and Paper Industry. The chips were oven-dried at 60 °C until a constant mass was reached, milled in a knife mill (Retsch SM 2000), and sieved to attain the 40–60 mesh fraction (particle size from 250 to 425 µm). After that, the extractive compounds were removed in a Soxhlet apparatus, with a sequence of dichloromethane, ethanol, and water under reflux for 6, 16, and 16 h, respectively, and dried.

The dioxane procedure was used to isolate the lignin from the eucalypt stump extractive-free material using 1,4-dioxane solvent (pure analysis (p.a.), Fisher Chemicals), according to Evtuguin et al. [26]. Around 10 g of dry extractive-free material was submitted to three sequential extractions with 200 mL dioxane/water solution (9:1, *v*/*v*) containing 0.1 M HCl (p.a. 37%, Fisher Chemicals), under a nitrogen atmosphere for 30, 40, and 40 min, respectively, followed by a fourth extraction using 200 mL dioxane/water solution (9:1, *v*/*v*) for 20 min. The liquid fractions were collected and concentrated in a rotary evaporator between extractions. The lignin in the concentrate was precipitated in distilled cold water (~1.0 L) and left overnight. The precipitated lignin was centrifuged in a Hermle Z326 at 4500 rpm for 10 min and washed with distilled water until the filtrates were of neutral pH. After that, the lignin was frozen at −80 °C and freeze-dried for 48 h. The powdered sample was used for further analyses. The yield of the dioxane lignin was about 60−65% of the total lignin content of the starting material (Klason lignin = 22.3%).

### 3.2. Kraft Lignin

Kraft lignin is industrial lignin and was obtained from a local cellulose and paper factory (Serbia) using black liquor from the kraft pulping process of poplar and beech wood (70:30) by precipitation with H_2_SO_4_ (p.a. 96%, Lach-ner). After precipitation, it was rinsed with distilled water, dried at room temperature, and milled to a fine powder (<0.25 mm).

### 3.3. Characterisation of the Lignins

#### 3.3.1. Analytical Pyrolysis (Py-GC/MS)

The eucalypt stump lignin and the kraft lignin were characterised by Py-GC/MS. Nearly 110 µg was weighted in a quartz boat and pyrolysed at 550 °C for 1 min in a 5150 CDS apparatus linked to a gas chromatograph (Agilent GC 7890B) with a mass detector (5977B) at 70 eV electron impact voltage. The volatiles were separated in the GC with a fused capillary column ZB-1701 (60 m × 0.25 mm i.d. × 0.25 µm film thickness). The carrier gas was helium at a total flow of 1 mL/min. The temperatures were: 280 °C (interface) and 270 °C (injector). The temperature program used was: 40 °C for 4 min, raised to 70 °C at a rate of 10 °C min^−1^, raised to 100 °C at 5 °C min^−1^, then to 265 °C at 3 °C min^−1^ (held for 3 min), and to 270 °C at 5 °C min^−1^ (held for 9 min). The compounds were identified by comparing with Wiley, NIST2014 computer libraries, and relevant literature [44,45]. The percentage of each compound was calculated based on the total area of the chromatogram. The percentage of *p*-hydroxyphenyl (H), guaiacyl (G), and syringyl (S) lignin-derived products were summed separately, and the S/G ratio and the S:G:H relation were calculated.

#### 3.3.2. Fourier Transform Infrared Spectroscopy (FTIR)

The FTIR spectra of the samples were recorded on a Thermo Nicolet iS20 FTIR spectrophotometer using a transmission technique in KBr pellets. For all spectra, 32 scans were recorded and averaged, with a resolution of 4 cm^−1^ for each spectrum. A DTGS detector was employed in the IR measurements. The baseline was corrected from 3800 to 700 cm^−1^ for comparison purposes.

#### 3.3.3. Elemental Analysis, Scanning Electron Microscopy (SEM), and Energy-Dispersive X-ray Spectroscopy (EDX) Analysis

The lignins (2–3 mg) were characterised by the percentage of carbon, hydrogen, nitrogen, and sulphur in an elemental analyser Thermo Finnigan-CE Instruments Flash EA 1112. The percentage of oxygen was calculated as [% O = 100% − (% C + % H + % N + % S)].

The eucalypt stump lignin and kraft lignin were also analysed by scanning electron microscopy (SEM) and energy-dispersive X-ray spectroscopy (EDX) using a JEOL JSM 6460LV Scanning Electron Microscope device and INCA X-sight (Oxford Instruments) software.

#### 3.3.4. Determination of the Point of Zero Charge (pH_pzc_)

The point of zero charge (pH_pzc_) was determined according to Smičiklas et al. [46]. In brief, 0.1 g of lignin was added to different reaction vessels containing 100 mL of 0.1 KNO_3_ (pure > 99%, Centrohem) solutions with pH from 1 to 12 and mixed for 24 h. The pH was adjusted using 0.1 M HNO_3_ (p.a. 65%, Lach-ner) and 0.1 M KOH. The adsorbent was then separated from the solution by filtration, and the final pH of the filtrates was measured. The pH_pzc_ was determined from the plateau of the plot pH_initial_ vs. pH_final_.

### 3.4. Batch Adsorption Experiments

Batch adsorption experiments were conducted on a laboratory shaker (MLW THYS-2) at room temperature by mixing 0.1 g of the investigated adsorbent and 100 mL of Cr(VI) aqueous solution, prepared from 0.25 moL/L K_2_Cr_2_O_7_ (p.a. > 99%, Centrohem) at pH 2, which was chosen according to the literature [47]. The contact time ranged from 10 min to 24 h, at an initial ion concentration of 50 mg/L for the kinetic studies. In contrast, the initial concentration varied from 10 to 1000 mg/L and the contact time was 24 h for the isotherm studies. After that, the adsorbent was separated from the aqueous solution by filtration, and the concentration of Cr(VI) ions in the solution initially (C_0_, mg/L) and at time t (C) were determined by oxidation–reduction titrations [48]. The adsorption capacity (q, mg/g) was calculated as follows:

(1) $$q=\frac{C_{0}-C}{m}$$

where m is the mass of added adsorbent in grams per litre of solution. All of the experiments were performed in duplicates.

Experimental data were fitted using well-known kinetic (pseudo-first [37], second-order [38], Elovich [39]) and isotherm models (Langmuir, Freundlich, Temkin, and Dubinin–Radushkevich [40]). The experiments were performed in duplicate.

## 4. Conclusions

This study demonstrated the potential for utilising eucalypt stump lignin extracted by the dioxane method to treat wastewater containing Cr(VI) ions. The dioxane method preserved the lignin structure and the monomeric composition with a predominant presence of S units (62%), followed by G units (21%), and a small number of H units (0.4%). In comparison, this distribution was 50%, 31%, and 2%, respectively, in the kraft lignin (used as reference). The eucalypt stump lignin is more homogenous than kraft lignin, which has flat layers and many pores suitable for ion binding. Even though adsorption experiments showed that both lignins removed almost all Cr(VI) ions present in the solution after 24 h of contact, the adsorption onto eucalypt stump lignin was faster in the first 8 h, which is a significant advantage for its practical application in water treatment. Kinetic studies showed that the pseudo-second-order model fitted the experimental data well for both investigated adsorbents and that the limiting factor of the adsorption rate of Cr(VI) ions was probably chemisorption. The adsorption of chromium ions by eucalypt stump lignin can be described very well by the Langmuir and Temkin models. In contrast, Langmuir’s model perfectly fitted kraft lignin adsorption. Both lignins showed very high efficiency for Cr(VI) ion removal, with the kraft lignin being slightly more efficient than the eucalypt stump lignin at or below the initial concentration of 50 mg/L, although the eucalypt stump lignin was more efficient at concentrations between 100 and 500 mg/L. Therefore, the eucalypt stump lignin is an interesting adsorbent, with a maximum adsorption capacity (q_m_) of 256 mg/g under the applied conditions compared with kraft lignin (q_m_ of 303 mg/g). 

The valorisation of forest waste is a fundamental step towards the zero-waste philosophy and the circular economy and biorefinery concepts. Considering this, further evaluation of eucalypt stump lignin as an adsorbent should include desorption studies and the possibility of multiple lignin usage in consecutive cycles of adsorption/desorption, as well as the management of loaded adsorbents. 

## Figures and Tables

**Table 1:** Identification and quantification of the compounds obtained by Py-GC/MS of the isolated lignins: *Eucalyptus* stump lignin and kraft lignin (% of the total area of the chromatogram).

Peak Number	RT	Compound	Origin	Eucalypt Lignin	Kraft Lignin
1	23.36	phenol	H	0.2	1.6
2	24.09	guaiacol	G	2.2	14.2
3	26.76	*p*-cresol	H	0.1	0.3
4	28.26	4-methylguaiacol	G	3.5	2.9
5	30.27	4-ethylphenol	H	0.1	0.1
6	31.74	4-ethylguaiacol	G	0.7	1.2
7	32.87	methylsyringol	S	0.1	0.4
8	34.13	4-vinylguaiacol	G	3.6	6.9
9	35.09	eugenol	G	0.1	0.3
10	35.20	4-propylguaiacol	G	0.2	0.2
11	36.32	syringol	S	6.5	30.7
12	36.78	isoeugenol	G	0.3	0.2
13	37.37	*cis* isoeugenol	G	0.4	0.3
14	39.43	*trans* isoeugenol	G	1.8	1.3
15	39.98	4-methylsyringol	S	8.5	5.4
16	40.46	vanillin	G	1.4	0.5
17	40.88	1-(4-hydroxy-3-methoxyphenyl)propyne	G	0.2	0.1
18	40.88	1-(4-hydroxy-3-methoxyphenyl)propyne	G	0.2	0.1
19	42.70	homovanillin	G	1.4	0.1
20	42.82	4-ethylsyringol	S	1.7	1.7
21	43.57	acetoguaiacone	G	1.1	0.9
22	45.06	4-vinylsyringol	S	6.1	5.2
23	45.48	guaiacylacetone	G	0.8	0.7
#24	45.71	4-allylsyringol	S	1.1	0.3
#25	45.71	4-propylsyringol	S	1.1	0.3
26	46.65	propioguaiacone	G	0.5	0.2
#27	47.17	coniferyl alcohol	G	0.8	0.1
#28	47.17	guaiacyl vinyl ketone	G	0.8	0.1
29	47.62	*cis* 4-propenylsyringol	S	1.2	0.4
30	48.94	4-propinylsyringol	S	1.0	0.0
31	49.25	4-propinylsyringol	S	0.6	0.3
32	49.14	1,6-anhydro-β-D-glucopyranose	Sugar	0.6	0.3
33	49.76	*trans* 4-propenylsyringol	S	5.9	1.7
34	50.01	dihydroconiferyl alcohol	G	0.1	0.0
35	50.97	syringaldehyde	S	6.2	0.8
36	52.45	homosyringaldehyde	S	3.6	0.1
37	53.05	syringic acid methyl ester	S	0.2	0.1
38	53.30	acetosyringone	S	4.2	1.5
39	54.58	*trans* coniferaldehyde	G	1.1	0.8
40	54.71	syringylacetone	S	2.6	0.2
41	55.76	propiosyringone	S	1.3	0.4
#42	56.30	syringyl vinyl ketone	S	1.4	0.1
#43	56.33	sinapyl alcohol isomer	S	1.9	0.1
44	62.87	*trans* sinapaldehyde	S	6.8	0.2
		**Total lignin (% of total area)**		83.5	83.1
		**Syringyl units (S)**		61.9	50.0
		**Guaiacyl units (G)**		21.1	31.1
		***p*** **-Hydroxyphenyl units (H)**		0.4	2.0
		**S/G ratio**		2.9	1.6
		**H:G:S**		1:50:146	1:16:26

RT—retention time (min); # overlapped compounds; Lignin derivatives: S—syringyl units; G—guaiacyl units; H—*p*-hydroxyphenyl units.

**Table 2:** Results of the elemental analysis of the isolated lignins (mass percentage).

Element	Eucalypt Stump Lignin	Kraft Lignin
C	58.2	60.5
H	5.8	5.1
N	0.0	0.0
S	0.0	2.0
O	36.0	32.5

**Table 3:** Parameters of kinetic and diffusion models for Cr(VI) adsorption onto eucalypt stump and kraft lignin.

Model	Equation	Parameter	Eucalypt Lignin	Kraft Lignin	Ref.
**Pseudo-first-order**	$\log\left(q_{e}-q_{t}\right)=\log q_{e}-\frac{k_{1}}{2.303}t$	q_e_ (mg/g)	50.00	50.00	[37]
		k_1_ (1/min)	0.00253	0.00276	
		R^2^	0.9626	0.944	
**Pseudo-second-order**	$\frac{t}{q_{t}}=\frac{1}{k_{2}\cdot q_{e}^{2}}+\left(\frac{1}{q_{e}}\right)\cdot t$	q_e_ (mg/g)	51.55	56.49	[38]
		k_2_ (g/mg min)	0.00835	0.0000915	
		R^2^	0.9995	0.9951	
**Elovich**	$q_{t}=\beta \ln\left(\alpha \beta \right)+\alpha \ln t$	α	5.45·10^6^	5.64⋅10^12^	[39]
		β	0.1112	0.0934	
		R^2^	0.9823	0.9507	

*q_e_* and *q_t_* (mg/g) are the amounts of chromium ions adsorbed per gram adsorbent at equilibrium and at time *t* (min); *k_1_* (L/min) is a pseudo-first-order rate constant, *k_2_* (g/mg min) is a pseudo-second-order rate constant, *α* (mg/g min) is the initial rate of adsorption, *β* (g/mg) is desorption constant. Ref.—reference.

**Table 4:** Isotherm models and corresponding parameters for chromium ions adsorption onto eucalypt stump lignin and kraft lignin.

Model	Equation	Parameter	Adsorbent		Ref.
			*Eucalyptus*	*Kraft*	
**Langmuir**	$\frac{C_{e}}{q_{e}}=\left(\frac{1}{q_{m}\cdot K_{L}}\right)+\left(\frac{1}{q_{m}}\right)\cdot C_{e}$	**q_m_ (mg/g)**	256.4	303.0	[40]
		K_L_	0.0385	0.0565	
		R^2^	0.9979	0.9997	
**Freundlich**	$lnq_{e}=lnK_{f}+\frac{1}{n}\cdot lnC_{e}$	K_F_	21.2	28.7	
		1/n	0.42	0.38	
		R^2^	0.8642	0.7065	
**Temkin**	$q_{e}=\frac{RT}{b}lnA_{T}+\frac{RT}{b}\;lnC_{e}$	b	3,098,523.9	3,098,523.8	
		A_T_	28282.5	126753.5	
		R^2^	0.9916	0.8634	
**Dubinin–Radushkevich**	$\ln q=ln\;q_{m}-A_{d}\;{\left(RT\ln\left(1+\frac{1}{C}\right)\right)}^{2}$	q_m_ (mg/g)	538.9	644.3	
		A_d_	−5 × 10^−9^	−5 × 10^−9^	
		R^2^	0.9141	0.7989	

q_e_ and q_m_ (mg/g) are equilibrium Cr(VI) concentrations per gram adsorbent, and a maximal adsorbed Cr(VI) per gram adsorbent, respectively; K_L_ (L/mg) is Langmuir constant, C_e_ (mg/L) is equilibrium Cr(VI) concentration in the solution, K_f_ is Freundlich constant (mg/g)/(l/mg)^1/n^, 1/n is constant related to the adsorbent surface heterogeneity, R is the ideal gas constant (8.314 J/mol K), T is the temperature (K). A_T_ (L/mg) is Temkin isotherm constant, and b (J/mol) is a constant related to the heat of adsorption, which is assumed to decrease linearly with the increase in surface coverage, A_d_ is the Dubinin–Radushkevich constant. Ref.—reference.

## Data Availability

The data attained during the current study are available from the corresponding author on reasonable request.

## Sample Availability

Samples of the compounds are not available from the authors.

## References

[1] Bortoluz J., Cemin A., Bonetto L.R., Ferrarini F., Esteves V.I., Giovanela M. (2019). Isolation, characterization and valorization of lignin from Pinus elliottii sawdust as a low-cost biosorbent for zinc removal. Cellulose.

[2] Lourenço A., Pereira H., Poletto M. (2018). Compositional Variability of Lignin in Biomass. Lignin—Trends and Applications.

[3] Ragauskas A.J., Beckham G.T., Biddy M.J., Chandra R., Chen F., Davis M.F., Davison B.H., Dixon R.A., Gilna P., Keller M. (2014). Lignin Valorization: Improving Lignin Processing in the Biorefinery. Science.

[4] Melro E., Filipe A., Sousa D., Medronho B., Romano A. (2020). Revisiting lignin: A tour through its structural features, characterization methods and applications. New J. Chem..

[5] Laurichesse S., Avérous L. (2014). Chemical modification of lignins: Towards biobased polymers. Prog. Polym. Sci..

[6] Yuan T.-Q., Xu F., Sun R.-C. (2012). Role of lignin in a biorefinery: Separation characterization and valorization. J. Chem. Technol. Biotechnol..

[7] Harmita H., Karthikeyan K., Pan X. (2009). Copper and cadmium sorption onto kraft and organosolv lignins. Bioresour. Technol..

[8] Ge Y., Li Z. (2018). Application of Lignin and Its Derivatives in Adsorption of Heavy Metal Ions in Water: A Review. ACS Sustain. Chem. Eng..

[9] Lalvani S.B., Hübner A., Wiltowski T.S. (2000). Chromium Adsorption by Lignin. Energy Sources.

[10] Lalvani S.B., Wiltowski T.S., Murphy D., Lalvani L.S. (1997). Metal Removal from Process Water by Lignin. Environ. Technol..

[11] Mohan D., Pittman C.U., Steele P.H. (2006). Single, binary and multi-component adsorption of copper and cadmium from aqueous solutions on Kraft lignin—A biosorbent. J. Colloid Interface Sci..

[12] Šćiban M.B., Klašnja M.T., Antov M.G. (2011). Study of the biosorption of different heavy metal ions onto Kraft lignin. Ecol. Eng..

[13] Cemin A., Ferrarini F., Poletto M., Bonetto L.R., Bortoluz J., Lemée L., Guégan R., Esteves V.I., Giovanela M. (2021). Characterization and use of a lignin sample extracted from Eucalyptus grandis sawdust for the removal of methylene blue dye. Int. J. Biol. Macromol..

[14] Jobby R., Jha P., Yadav A.K., Desai N. (2018). Biosorption and biotransformation of hexavalent chromium [Cr(VI)]: A comprehensive review. Chemosphere.

[15] Zhang H., Zhou H. (2022). Industrial lignins: The potential for efficient removal of Cr(VI) from wastewater. Environ. Sci. Pollut. Res..

[16] Zhang H., Tian Y., Niu Y., Dong X., Lou H., Zhou H. (2022). Lignosulfonate/N-butylaniline hollow microspheres for the removal of Cr(VI): Fabrication, adsorption isotherm and kinetics. J. Water Process Eng..

[17] Tian Y., Zhou H. (2022). A novel nitrogen-doped porous car bon derived from black liquor for efficient removal of Cr(VI) and tetracycline: Comparison with lignin porous carbon. J. Clean. Prod..

[18] Pakade V.E., Tavengwa N.T., Madikizela L.M. (2019). Recent advances in hexavalent chromium removal from aqueous solutions by adsorptive methods. RSC Adv..

[19] Netzahuatl-Muñoz A.R., Cristiani-Urbina M.D.C., Cristiani-Urbina E. (2015). Chromium Biosorption from Cr(VI) Aqueous Solutions by Cupressus lusitanica Bark: Kinetics, Equilibrium and Thermodynamic Studies. PLoS ONE.

[20] Tejada-Tovar C., Villabona-Ortíz A., Ortega-Toro R., Mancilla-Bonilla H., Espinoza-León F. (2021). Potential Use of Residual Sawdust of *Eucalyptus globulus* Labill in Pb (II) Adsorption: Modelling of the Kinetics and Equilibrium. Appl. Sci..

[21] Gominho J., Lourenço A., Miranda I., Pereira H. (2012). Chemical and fuel properties of stumps biomass from Eucalyptus globulus plantations. Ind. Crop. Prod..

[22] Gominho J., Lopes C., Lourenço A., Simões R., Pereira H. (2014). Eucalyptus globulus Stumpwood as a Raw Material for Pulping. BioResources.

[23] Gominho J., Costa R., Lourenco A., Neiva D., Pereira H. (2019). The effect of different pre-treatments to improve delignification of eucalypt stumps in a biorefinery context. Bioresour. Technol. Rep..

[24] Pinto F., Gominho J., André R.N., Gonçalves D., Miranda M., Varela F., Neves D., Santos J., Lourenço A., Pereira H. (2017). Improvement of gasification performance of Eucalyptus globulus stumps with torrefaction and densification pre-treatments. Fuel.

[25] Luís A., Neiva D.M., Pereira H., Gominho J., Domingues F., Duarte A.P. (2016). Bioassay-guided fractionation, GC–MS identification and in vitro evaluation of antioxidant and antimicrobial activities of bioactive compounds from Eucalyptus globulus stump wood methanolic extract. Ind. Crop. Prod..

[26] Evtuguin D.V., Neto C.P., Silva A.M.S., Domingues P.M., Amado F.M.L., Robert D., Faix O. (2001). Comprehensive Study on the Chemical Structure of Dioxane Lignin from Plantation *Eucalyptus globulus* Wood. J. Agric. Food Chem..

[27] Lourenço A., Gominho J., Pereira H., Kusch P. (2019). Chemical Characterisation of Lignocellulosic Materials by Analytical Pyrolysis. Analytical Pyrolysis.

[28] Wen J.-L., Sun S.-L., Yuan T.-Q., Sun R.-C. (2014). Structural elucidation of whole lignin from Eucalyptus based on preswelling and enzymatic hydrolysis. Green Chem..

[29] Boeriu C.G., Bravo D., Gosselink R.J., van Dam J.E. (2004). Characterisation of structure-dependent functional properties of lignin with infrared spectroscopy. Ind. Crop. Prod..

[30] Kubo S., Kadla J.F. (2005). Hydrogen Bonding in Lignin: A Fourier Transform Infrared Model Compound Study. Biomacromolecules.

[31] Faix O. (1991). Classification of lignins from different botanical origins by FT-IR spectroscopy. Holzforschung.

[32] Gabov K., Gosselink R.J.A., Smeds A.I., Fardim P. (2014). Characterization of Lignin Extracted from Birch Wood by a Modified Hydrotropic Process. J. Agric. Food Chem..

[33] De Meester B., Calderón B.M., De Vries L., Pollier J., Goeminne G., Van Doorsselaere J., Chen M., Ralph J., Vanholme R., Boerjan W. (2020). Tailoring poplar lignin without yield penalty by combining a null and haploinsufficient CINNAMOYL-CoA REDUCTASE2 allele. Nat. Commun..

[34] Hansen B., Kusch P., Schulze M., Kamm B. (2016). Qualitative and Quantitative Analysis of Lignin Produced from Beech Wood by Different Conditions of the Organosolv Process. J. Polym. Environ..

[35] Zikeli F., Vinciguerra V., Taddei A.R., D’Annibale A., Romagnoli M., Mugnozza G.S. (2018). Isolation and characterization of lignin from beech wood and chestnut sawdust for the preparation of lignin nanoparticles (LNPs) from wood industry side-streams. Holzforschung.

[36] Fiol N., Villaescusa I. (2008). Determination of sorbent point zero charge: Usefulness in sorption studies. Environ. Chem. Lett..

[37] Ofomaja A.E. (2010). Intraparticle diffusion process for lead(II) biosorption onto mansonia wood sawdust. Bioresour. Technol..

[38] Ho Y.S., Wase D.A.J., Forster C.F. (1995). Batch nickel removal from aqueous solution by sphagnum moss peat. Water Res..

[39] Zeldowitsch J. (1934). Uber den mechanismus der katalytischen oxydationvon CO and MnO2. Acta Physicochim..

[40] Ayawei N., Ebelegi A.N., Wankasi D. (2017). Modelling and Interpretation of Adsorption Isotherms. J. Chem..

[41] Gupta G.K., Mondal M.K. (2020). Mechanism of Cr(VI) uptake onto sagwan sawdust derived biochar and statistical optimization via response surface methodology. Biomass Convers. Biorefinery.

[42] (2009). Vinodhini V, Das N Mechanism of Cr(VI) biosorption by neem sawdust. Am.-Eurasian J. Sci. Res..

[43] Suksabye P., Thiravetyan P. (2012). Cr(VI) adsorption from electroplating plating wastewater by chemically modified coir pith. J. Environ. Manag..

[44] Faix O., Fortman I., Bremer J., Meier D. (1991). Thermal degradation products of wood. Gas chromatographic separation and mass spectrometric characterisation of lignin derived products. Holz als Roh-und Werkstoff.

[45] Ralph J., Hatfield R.D. (1991). Pyrolysis-GC-MS characterization of forage materials. J. Agric. Food Chem..

[46] Smičiklas I., Milonjić S., Pfendt P., Raičević S. (2000). The point of zero charge and sorption of cadmium (II) and strontium (II) ions on synthetic hydroxyapatite. Sep. Purif. Technol..

[47] Albadarin A.B., Al-Muhtaseb A.H., Al-Laqtah N.A., Walker G.M., Allen S.J., Ahmad M.N. (2011). Biosorption of toxic chromium from aqueous phase by lignin: Mechanism, effect of other metal ions and salts. Chem. Eng. J..

[48] Lide D.R. (2006). CRC Handbook of Chemistry and Physics.

